# Angiotensin II Facilitates Fibrogenic Effect of TGF-β1 through Enhancing the Down-Regulation of BAMBI Caused by LPS: A New Pro-Fibrotic Mechanism of Angiotensin II

**DOI:** 10.1371/journal.pone.0076289

**Published:** 2013-10-14

**Authors:** Yu-Sheng Li, Shu-Yuan Ni, Ying Meng, Xiao-Lan Shi, Xu-Wen Zhao, Hai-Hua Luo, Xu Li

**Affiliations:** 1 Department of Pathophysiology, Southern Medical University, Guangzhou, China; 2 Department of ICU, The Third Affiliated Hospital of Guangzhou Medical University, Guangzhou, China; 3 Department of Respiratory Diseases, Southern Medical University, Nanfang Hospital, Guangzhou, China; 4 Department of Emergency, Southern Medical University, Nanfang Hospital, Guangzhou, China; Institute for Nutritional Sciences, China

## Abstract

Angiotensin II has progressively been considered to play an important role in the development of liver fibrosis, although the mechanism isn't fully understood. The aim of this study was to investigate a possible pro-fibrotic mechanism, by which angiotensin II would enhance the pro-fibrotic effect of transforming growth factor beta 1 (TGF-β1) through up-regulation of toll-like receptor 4 (TLR4) and enhancing down-regulation of TGF-β1 inhibitory pseudo-receptor—BAMBI caused by LPS in hepatic stellate cells (HSCs). Firstly, the synergistic effects of angiotensin II, TGF-β1 and LPS on collagen 1α production were confirmed *in vitro* by ELISA, in which angiotensin II, LPS and TGF-β1 were treated sequentially, and *in vivo* by immunofluorescence, in the experiments single or multiple intra-peritoneally implanted osmotic mini-pumps administrating angiotensin II or LPS combined with intra-peritoneal injections of TGF-β1 were used. We also found that only LPS and TGF-β1 weren't enough to induce obvious fibrogenesis without angiotensin II. Secondly, to identify the reason of why angiotensin II is so important, the minute level of TLR4 in activated HSCs - T6 and primary quiescent HSCs of rat, up-regulation of TLR4 by angiotensin II and blockage by different angiotensin II receptor type 1 (AT1) blockers in HSCs were assayed by western blotting *in vitro* and immunofluorescence *in vivo*. Finally, BAMBI expression level, which is regulated by LPS-TLR4 pathway, was detected by qRT-PCR and results showed angiotensin II enhanced the down-regulation of *BAMBI* mRNA caused by LPS *in vitro* and *in vivo*, and TLR4 neutralization antibody blocked this interactive effect. These data demonstrated that angiotensin II enhances LPS-TLR4 pathway signaling and further down-regulates expression of BAMBI through up-regulation of TLR4, which results in facilitation of pro-fibrotic activity of TGF-β1. Angiotensin II, LPS and TGF-β1 act synergistically during hepatic fibrogenesis, showing crosstalks between angiotensin II-AT1, LPS-TLR4 and TGF-β1-BAMBI signal pathways in rat HSCs.

## Introduction

Many kinds of growth factors, vasoactive substances, inflammatory cytokines and adipokines are proved to be associated with hepatic fibrogenesis [1]–[3]. Transforming growth factor-beta 1 (TGF-β1), platelet-derived growth factor (PDGF) and angiotensin II (Ang II) are the key pro-fibrotic agents. However, a single factor only exhibits mild to moderate pro-fibrotic activity in transgenic mice [4]–[7]. That liver fibrosis can be regressed has been confirmed by many clinical and experimental studies [8], [9], although the effects of single-targeted anti-fibrogenic therapies are unsatisfactory [8], [10]. These facts indicate that the essences of hepatic fibrogenesis mechanisms remain uncovered. However, the research before, which mainly focuses on one factor or single signal pathway, dose not reflect the event occurs *in vivo*.

During the process of chronic liver inflammation, activated macrophages [11], [12] and hepatic stellate cells (HSCs) [13] release a lot of PDGF. The PDGF-receptor alpha (PDGFRα) is converted to PDGFRβ during activation [14], which mediates proliferation of HSCs [15]. Each member of the renin-angiotensin system (RAS) expresses itself in the fibrotic liver [16]. Especially, the angiotensin converting enzyme (ACE) and chymase are up-regulated [17]. Activated myofibroblastic HSCs express the elements of RAS and produce Ang II. Moreover, a lot of systemic Ang II is delivered into the liver during fibrogenesis [3]. Also activated angiotensin II receptor type 1 (AT1) expresses on the surface of HSCs and assembles into hepatic fibrotic area [18], [19]. In addition, TGF-β1 is up-regulated in activated Kupffer's cells (KCs) and HSCs [20], while HSCs express TGF-β1 receptors (TGFβ1R) constitutively [21]. In summary, not only are all kinds of pro-fibrogenic molecules present in the microenvironment of the fibrotic liver, but also are their receptors expressed in HSCs. Therefore, HSC receives stimulations from all kinds of pro-fibrotic molecules and the subsequent signal pathways are integrated intra-cellularly. The final interactive effect may be positive or negative.

Seki E, et al. proved that intestinal LPS, which is increased in fibrotic liver, doesn't induce collagen 1α (Col 1α) synthesis in HSCs directly, but can down-regulate BMP and activin membrane-bound inhibitor homolog (BAMBI), which is a pseudo-receptor of TGF-β1, through the toll-like receptor 4 (TLR4), thereby facilitating the pro-fibrogenic function of TGF-β1 [22]. These findings not only indicate a crosstalk between inflammatory and pro-fibrogenic signal pathways, but also suggest a crosstalk between two kinds of pro-fibrogenic factors, TGF-β1 and Ang II. The reason to believe so is that Ang II can induce TLR4 expression and enhance the activity of the LPS-TLR4 signal pathway in many kinds of cells, such as mouse mesangial cells [23], murine podocytes [24], human smooth muscle cells [25] and rat peritoneal mesothelial cells [26]. Therefore, we suggest that Ang II can up-regulate TLR4 in HSCs and enhance the function of LPS in the fibrotic liver. Finally, it may facilitate the pro-fibrogenic function of TGF-β1 through enhancing down-regulation of BAMBI. In this paper, we report for the first time that Ang II up-regulates TLR4 protein expression through AT1, enhances the down-regulation of *BAMBI* mRNA caused by LPS and facilitates the pro-fibrogenic function of TGF-β1 in HSCs.

## Materials and Methods

### Reagents

Unless otherwise stated, all reagents used purchased from Sigma (St. Louis, MO, USA).

### Cell Culture and Treatment

**Figure 1:**
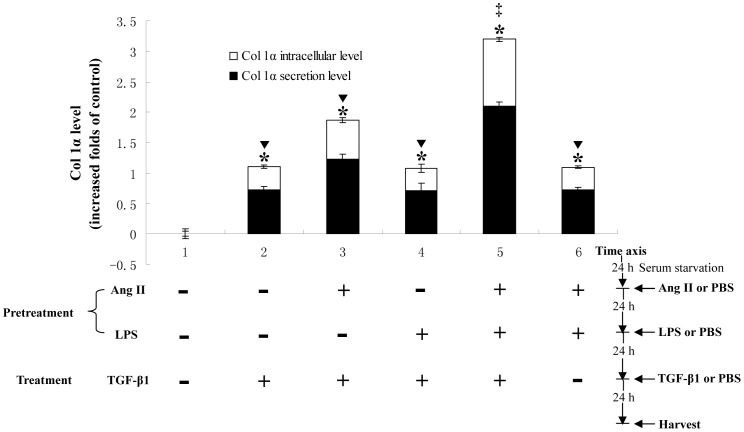
Ang II, LPS and TGF-β1 show synergistic effects on Col 1α production *in vitro*. 90% confluent T6 cells were treated as described in ***Materials and methods*** after serum starvation for 24 h. The spent media and total protein of each group cells were harvested for measuring Col 1α level using ELISA. The results are representative of three separate experiments. * *p*<0.05, significantly up-regulated Col 1α levels in all treated groups compared with P (-, -) T (-) group. ▾ *p*<0.05, the Col 1α levels of P (-, -) T (TGF-β1), P (Ang II, -) T (TGF-β1), P (-, LPS) T (TGF-β1) and P (Ang II, LPS) T (-) groups are lower than that of P (Ang II, LPS) T (TGF-β1) group. ‡ *p*<0.05 shows synergistic effects between Ang II, LPS and TGF-β1 stimulation using factorial analysis.

An immortalized rat HSC line—T6 cells (China Center for Type Culture Collection, CCTCC, Wuhan, China), recognized as fully activated HSCs with biological behaviour similar to primary HSCs [27], [28], were used *in vitro* studies. The cells were initially serum starved for 24 h and then treated with various concentrations (0, 10^−8^, 10^−7^, 10^−6^, 10^−5^ mol/L) of Ang II for 24 h or with 10^−6^ mol/L Ang II for various periods of time (0, 12, 24, 48 h) to determine the up-regulation of Tlr4. Then, cells were exposed to various concentrations (0, 10^−8^, 10^−7^, 10^−6^, 10^−5^ mol/L) of Irbesartan (Irb), which is a specific AT1 blocker, for 1 h followed by Ang II (10^−6^ mol/L) for 24 h to make sure that Ang II induced Tlr4 expression through AT1. Finally, T6 cells were treated as described in the related result figure to analyze whether Ang II enhanced the activity of LPS-TLR4 signal pathway, which was done by assaying levels of *BAMBI mRNA*. And also, anti-TLR4 neutralization antibody (1 mg/ml) (abcam, Cambridge, MA, USA) was used to confirm that the enhancement caused by Ang II was through up-regulated TLR4. Irrelevant IgG2a (1 mg/ml) (R&D Systems, Minneapolis, MN, USA) was used as control. In the experiment on the synergistic effects of Ang II, LPS and TGF-β1 on Col 1α production *in vitro*, cells were grouped as follows: Pretreatment (P) (-, -) Treatment (T) (-), P (-, -) T (TGF-β1), P (Ang? II, -) T (TGF-β1), P (-, LPS) T (TGF-β1), P (Ang II, LPS) T (TGF-β1), P (Ang II, LPS) T (-). For a detailed treatment protocol, see time axis in the result figure 1. The concentrations of Ang II, LPS and TGF-β1 are 10^−6^ mol/L, 100 ng/ml and 300 pg/ml, respectively. Cells were washed once with PBS before changing stimulation. Cells and cultured supernatant were harvested after completed exposure.

### Primary HSCs isolation

Primary HSCs were isolated using a published protocol [29].

### In Vivo Models of Rat Liver Fibrosis and treatments

It is easily to establish liver fibrosis rat models by carbon tetrachloride (CCl_4_) subcutaneous injection, dimethylnitrosamine (DMN) intraperitoneal injection or bile duct ligation (BDL), and the levels of Ang II, LPS and TGF-β1 are similarly up-regulated in the fibrotic livers of these rat models [3], [22]. Under these conditions, however, crosstalks between Ang II and TGF-β1, bridged by the LPS-TLR4 signal pathway, could not be easily distinguished. Therefore, single or mutiple osmotic mini-pumps (ALZET, Cupertino, CA, USA) were implanted intra-peritoneally, and filled with Ang II [30], LPS [31] or PBS, were used to reproduce the in-vivo experiment. In pump model, the reagents would accumulate into liver through the portal vein, and first take effect or be degraded there. The dose and rate of reagents used were determined by preliminary experiments to optimally reduce systemic effects.

**Figure 2:**
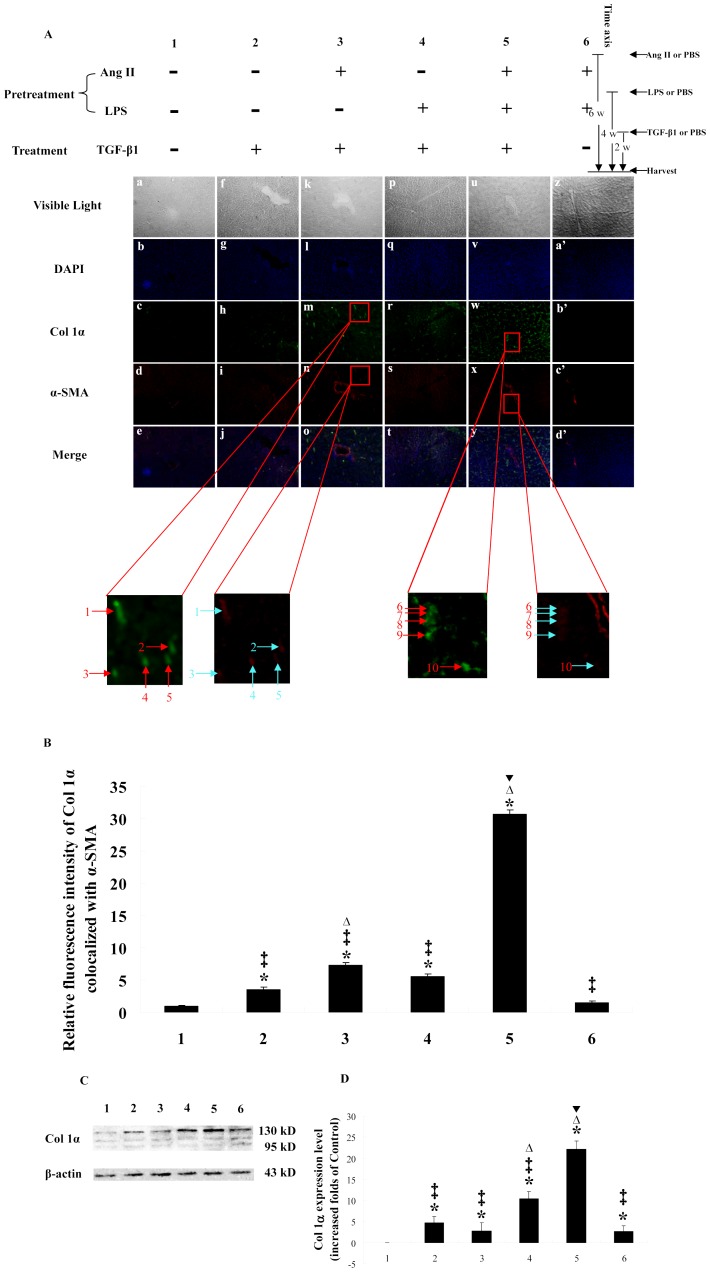
Ang II, LPS and TGF-β1 show synergistic effect on Col 1α production *in vivo*. Rats of each group were treated as shown in the figure. A: Hepatic cryo-sections of each rat show blue-stained nuclei, while Col 1α (green) and α-SMA (red) are demonstrated by immunofluorescence (X 100). B: Bar-graph of relative fluorescence intensity of Col 1α co-localized with α-SMA. C: Western blotting results of rat liver homogenates. D: Bar-graph of the densities of western blotting. The density of each band was measured using a densitometer, and the density of each Col 1α band was normalized using the density of its corresponding β-actin band. The results are representative of 3 separate experiments. * *p*<0.05, significantly up-regulates Col 1α expression level in 2, 3, 4, 5 and 6 group compared with group1. ‡ *p*<0.05, the Col 1α expression levels are lower than that of group 5. Δ *p*<0.05, the Col 1α expression levels are higher than that of group 2. ▾ *p*<0.05 shows a synergistic effect between Ang II, LPS and TGF-β1 stimulation using factorial analysis. “→” or “↑” show the co-localization of Col 1α and α-SMA (X 500).

Male Wistar rats weighing 220–280 g were purchased from the Laboratory Animal Center of Southern Medical University, Guangzhou, China, and all animal experiments were approved by the Committee on the Ethics of Animal Experiments of Southern Medical University. In experiment on Ang II up-regulating Tlr4 *in vivo*, 24 rats were randomly separated into three groups [Ang II (100 ng/kg/min), LPS (100 ng/kg/d), PBS and Ang II (100 ng/kg/min) + losartan (50 mg/kg/d)]. Then the osmotic pumps with Ang II, LPS or PBS were implanted intra-peritoneally. The pumps released their contents at a stable rate for 6 weeks. For Ang II (100 ng/kg/min) + losartan (50 mg/kg/d) group, losartan was given by intragastric gavage daily. Finally, the rats were killed and livers were collected after irrigation. In the experiment of the synergistic effects of Ang II, LPS and TGF-β1 on Col 1α production *in vivo*, 54 rats were randomly separated into: P (-, -) T (-), P (-, -) T (TGF-β1), P (Ang II, -) T (TGF-β1), P (-, LPS) T (TGF-β1), P (Ang II, LPS) T (TGF-β1), P (Ang II, LPS) T (-). See time axis in the result figure 2 for details of treatment procedure for each group. Two osmotic pumps were used for pretreatments (pump 1 with Ang II (100 ng/kg/min) or PBS for 6 weeks, and pump 2 with LPS (100 ng/kg/d) or PBS, added 2 weeks later, and operated for another 4 weeks), in combination with intra-peritoneal injections of TGF-β1 (300 µg/kg/d) [32] or PBS for 2 weeks. After pretreatment, 3 rats of each group were killed, and primary HSCs were isolated for checking the expression level of Tlr4 and *BAMBI mRNA*. After treatment, the left liver lobes were collected for immunofluorescence and the right one for immunoblotting.

### Tlr4 and Col 1α western blotting

Using the protocol published before [33] with primary antibody of Tlr4 (Santa Cruz, Santa Cruz, CA, USA) or Col 1α (Sigma, St. Louis, MO, USA) followed by secondary antibodies incubation. Detect the probing protein with SuperSignal West Pico Chemiluminescent Substrate (Thermo Scientific, Waltham, MA, USA).

### Immunofluorescence

For immunofluorescence, liver cryo sections were incubated with antibodies to alpha smooth muscle actin (α-SMA) (abcam, Cambridge, MA, USA) and TLR4 (abcam, Cambridge, MA, USA) or antibodies to α-SMA and Col 1α (Sigma, St. Louis, MO, USA).

### Quantitative realtime-polymerase chain reaction (qRT-PCR)

T6 cells or rat livers were harvested and total RNA was extracted using the Trizol reagent kit (Takara, Otsu, Shiga, Japan). 100 ng total RNA was used for qRT-PCR with iScript One-Step RT-PCR Kit (Bio-Rad, Hercules, CA, USA) according to the manufacturer's instructions. *BAMBI* forward primer: 5′-TGAAGCCTCAGGACAAGGAAA-3′; reverse primer: 5′-ACGGAACCACAGTTCTTTGGA-3′; *GAPDH* forward primer: 5′-CATTGTTGCCATCAACGACC-3′; reverse primer: 5′-TCACACCCATCACAAACATG-3′; (IDT, Coralville, IA, USA). The levels of *BAMBI* transcripts were normalized to *GAPDH* transcripts using the 2^−△△Ct^ method.

### ELISA

The full sample preparation protocol can be found in an earlier work [33]. In the direct ELISA for Col 1α, primary antibody to Col 1α, goat anti-mouse IgG and peroxidase-labeled streptavidin antibody (KPL, Gaithersburg, MD, USA) were used. A standard ELISA curve was generated for each plate.

### Statistical analysis

All data are presented as means ± SD and analyzed with SPSS 13.0. The differences between different groups were assessed by two-way ANOVA and *p*<0.05 were regarded significant. The interactive effects between different factors were analyzed by factorial analysis.

## Results

### Ang II, LPS and TGF-β1 show synergistic effects on Col 1α production in vitro

As shown in Fig. 1, P (-, -) T (TGF-β1), P (Ang II, -) T (TGF-β1), P (-, LPS) T (TGF-β1), P (Ang II, LPS) T (TGF-β1) and P (Ang II, LPS) T (-) increased Col 1α secretion (0.72 ± 0.05, 1.22 ± 0.08, 0.71 ± 0.13, 2.10 ± 0.07 and 0.72 ± 0.05 folds, respectively) and intracellular level (0.38 ± 0.02, 0.64 ± 0.04, 0.37 ± 0.07, 1.10 ± 0.04 and 0.38 ± 0.02 folds, respectively) compared with P (-, -) T (-) group. There was no difference between the P (-, -) T (TGF-β1) group and the P (-, LPS) T (TGF-β1) group (secretion: 0.72 ± 0.05 vs. 0.71 ± 0.13 folds, intracellular levels: 0.38 ± 0.02 vs. 0.37 ± 0.07 folds), showing that LPS did not induce Col 1α secretion, which was in accord with a previous study [22]. Moreover, there was no synergistic effect between LPS and TGF-β1, because there might be only trace amounts of TLR4 in HSCs without Ang II, and down-regulation of BAMBI was limited. Compared the results between P (Ang II, LPS) T (TGF-β1) and P (-, -) T (TGF-β1) together with P (Ang II, LPS) T (-), Ang II, LPS and TGF-β1 show synergistic effects on Col 1α production (F = 58.974, P<0.05). The Col 1α levels of P (Ang II, LPS) T (TGF-β1) group were higher than that of P (-, LPS) T (TGF-β1) group, showing that the synergistic effect might need the up-regulation of TLR4 induced by Ang II. Meanwhile, the Col 1α levels of P (Ang II, LPS) T (TGF-β1) group were higher than that of P (Ang II, -) T (TGF-β1) group, showing that the crosstalk between Ang II and TGF-β1 is bridged by LPS-TLR4 signal pathway.

### Ang II, LPS and TGF-β1 show synergistic effects on Col 1α production in vivo

To reproduce the above in-vitro experiments *in vivo*, we used intra-peritoneally implanted osmotic pumps. The Col 1α levels of P (Ang II, -) T (TGF-β1) (Fig. 2A k-o), P (-, LPS) T (TGF-β1) (Fig. 2A p-t), P (Ang II, LPS) T (TGF-β1) (Fig. 2A u-y) were up-regulated compared with the P (-, -) T (-) group (Fig. 2A a-e), which is in accord with the western blots (2.72 ± 1.96, 10.42 ± 1.64 and 22.16 ± 1.89 folds, respectively, Fig. 2 C and D). No such up-regulations were found for P (-, -) T (TGF-β1) (Fig. 2A f-j) or for P (Ang II, LPS) T (-) (Fig. 2A z-d′), although a pro-fibrogenic effect was found by western blotting (4.67 ± 1.44 and 2.66 ± 1.25 folds, respectively, Fig. 2 C and D). By comparing the results between P (Ang II, LPS) T (TGF-β1) and P (-, -) T (TGF-β1) together with P (Ang II, LPS) T (-), we concluded that Ang II, LPS and TGF-β1 act synergistically as same as in-vitro study. LPS didn't directly induce any Col 1α production in HSCs (Fig. 1). Therefore, the synergistic effect between Ang II and TGF-β1 is dependent on a bridge-like function of the LPS-TLR4 signal pathway through the comparation between P (Ang II, LPS) T (TGF-β1) and P (Ang II, -) T (TGF-β1). The key step of the crosstalk between Ang II and TGF-β1, bridged by the LPS-TLR4 signal pathway *in vivo*, was the up-regulation of TLR4 expression on HSCs by Ang II, concluded by comparing P (-, LPS) T (TGF-β1) with P (Ang II, LPS) T (TGF-β1). The difference described above may be due to the lack of TLR4 expression on the surface of HSCs in the absence of Ang II. It is, therefore, demonstrated that the synergistic effects between Ang II and TGF-β1 during hepatic fibrosis doesn't only require the LPS-TLR4 signal pathway, but also up-regulation of HSC TLR4 expression by Ang II. The western blots (Fig. 2 C and D) showed that the Col 1α levels of P (-, LPS) T (TGF-β1) are higher than those of P (-, -) T (TGF-β1) and P (Ang II, -) T (TGF-β1) (10.42 ± 1.64 vs. 4.67 ± 1.44 and 2.72 ± 1.96 folds), which are values different from the *in vitro* study. The reason for this discrepancy may be that perhaps not only HSCs but also KCs can produce Col 1α as a response to LPS [34]. The changes of the TLR4 and *BAMBI mRNA* expressions in individual rats were assayed to confirm the effectiveness of Ang II and LPS stimulation (data not show).

### Ang II induces HSC Tlr4 expression in dose- and time-dependent manner

**Figure 3:**
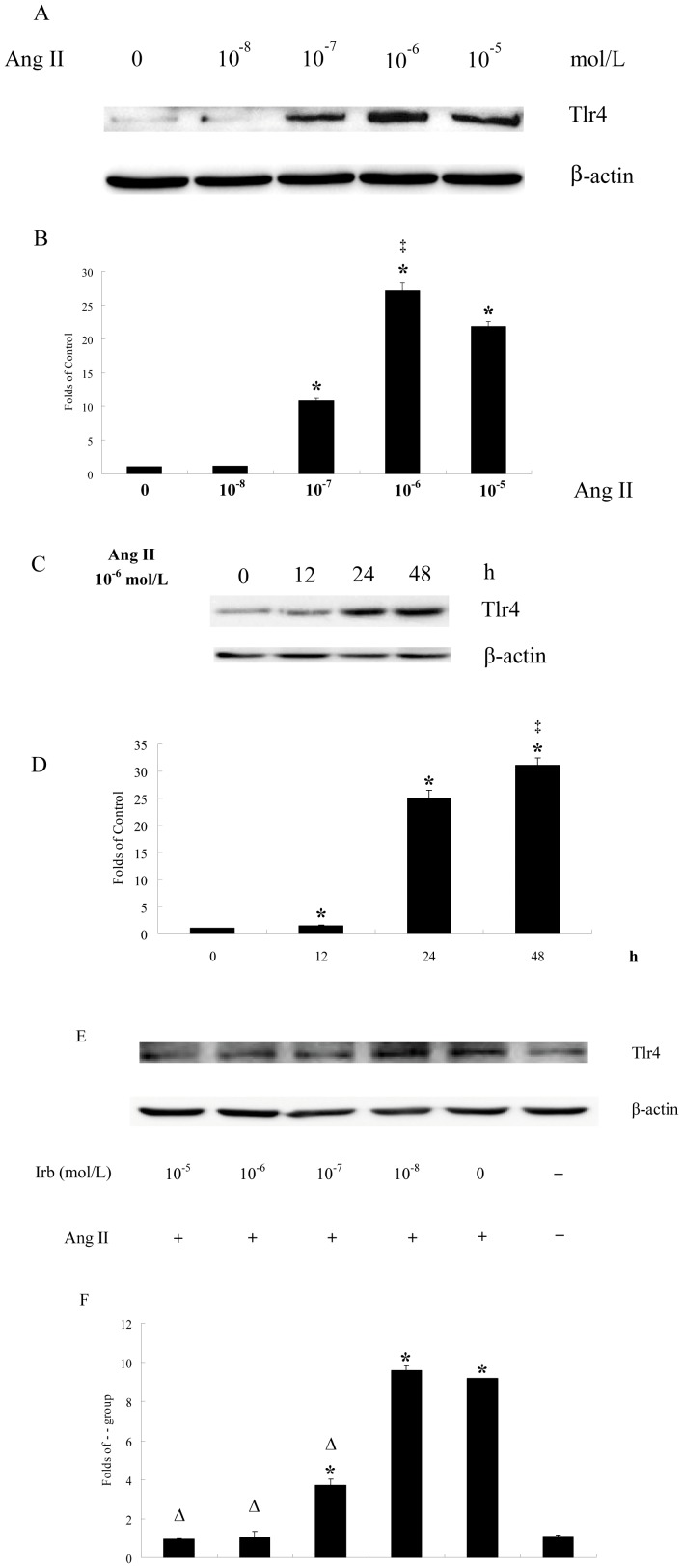
Ang II induces Tlr4 expression in dose- and time-dependent manner that is blocked by ARB. 90% confluent T6 cells were treated with Ang II (0, 10^−8^, 10^−7^, 10^−6^, 10^−5^ mol/L) for 24 h or Ang II 10^−6^ mol/L for 0, 12, 24, 48 h after serum starvation for 24 h. In the ARB blocking experiment, 90% confluent T6 cells were pretreated with Irb (0, 10^−8^, 10^−7^, 10^−6^, 10^−5^ mol/L) for 1 h after a 24 h serum starvation period. Then T6 cells were treated with Ang II (10^−6^ mol/L) for another 24 h. Cells were then harvested and total protein was collected. Equal protein aliquots of cell lysate were examined by immunoblotting with antibodies against Tlr4 or β-actin. β-actin was used to verify equal gel loading and trans-blot efficiencies. A, C and E: Results of western blots. B, D and F: Bar-graph of the densities of western blots. * *p*<0.05, Ang II significantly up-regulated Tlr4 as compared with the control group. ‡ *p*<0.05, the peak appears. Δ *p*<0.05, the Tlr4 expression levels are lower than that of Ang II 10^−6^ mol/L group.

The maximal pro-fibrotic effect of TGF-β1 needs both of Ang II and LPS proved above may be because HSCs express insufficient TLR4 without Ang II. To test whether Ang II induced TLR4 expression in HSCs, fully activated rat HSC T6 cells were chosen. As shown in Fig. 3 A-D, activated rat HSCs expressed only low levels of Tlr4 before Ang II stimulation, but it was then up-regulated by Ang II in dose-dependent manner [10^−7^ mol/L (10.8 ± 0.38 folds), 10^−6^ mol/L (27.2 ± 1.25 folds), 10^−5^ mol/L (21.9 ± 0.70 folds)] and time-dependent manner [12 h (1.50 ± 0.17 folds), 24 h (25.0 ± 1.49 folds), 48 h (31.0 ± 1.37 folds)]. The peak values appeared at 10^−6^ mol/L and after exposure for 48 h.

### AT1R antagonist blocks Ang II-mediated up-regulation of Tlr4 dose-dependently in vitro

Irb was used to inhibit interaction between Ang II and AT1. Expression of HSC Tlr4 was up-regulated by Ang II (10^−6^ mol/L) (9.17 ± 0.26 folds of - - group). Irb (10^−7^ mol/L) inhibited this up-regulation (3.70 ± 0.23 folds vs. 9.17 ± 0.26 folds). Irb (10^−6^ mol/L or 10^−5^ mol/L) blocked totally (1.03 ± 0.03 folds or 0.97 ± 0. 07 folds vs. 1.06 ± 0.04 folds, respectively), showing that, AT1R antagonist blocks Tlr4 up-regulation by Ang II dose-dependently. Ang II obviously up-regulates Tlr4 expression solely through AT1 since Irb prevents it (Fig. 3 E and F).

### HSC Tlr4 expression is up-regulated by Ang II and blocked by ARB in vivo

**Figure 4:**
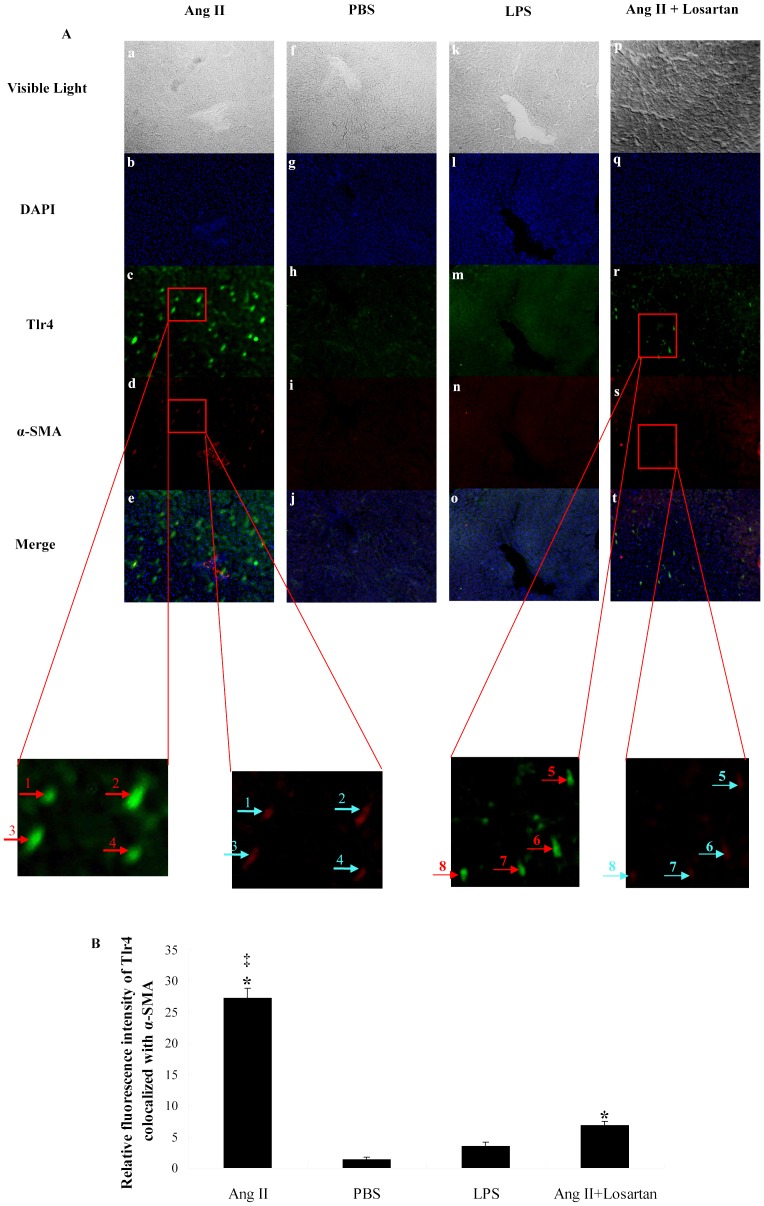
Tlr4 expression of activated HSCs is up-regulated by Ang II and blocked by ARB in rat liver. Rats of each group were treated as described in Materials and Methods. A: Hepatic cryo-sections of each rat show blue-stained nuclei, while Tlr4 (green) and α-SMA (red) are demonstrated by immunofluorescence (X 100). B: Bar-graph of relative fluorescence intensity of Tlr4 co-localized with α-SMA. * *p*<0.05, Ang II significantly up-regulated Tlr4 expression level as compared with PBS. ‡ *p*<0.05, the Tlr4 expression level of Ang II treated groups are higher than that of Ang II + Losartan groups. “→” shows the co-localization of Tlr4 and α-SMA (X 500).

Next, we examined, using immunofluorescence, whether HSC Tlr4 expression is up-regulated by Ang II and blocked by ARB (Losartan) *in vivo*. In Fig. 4, Tlr4 is shown in green, and α-SMA, which is the marker of activated HSCs, in red. According to the merge pictures, Tlr4 expression of activated HSCs is up-regulated by persistent Ang II intra-peritoneal administration, using osmotic pumps (Fig. 4A a-e) and blocked by intra-gastric administration of losartan (Fig. 4A p-t). The up-regulation of Tlr4 could not be found in PBS treatment group or LPS treatment group.

### Ang II enhances the HSC LPS-TLR4 signal pathway activity

**Figure 5:**
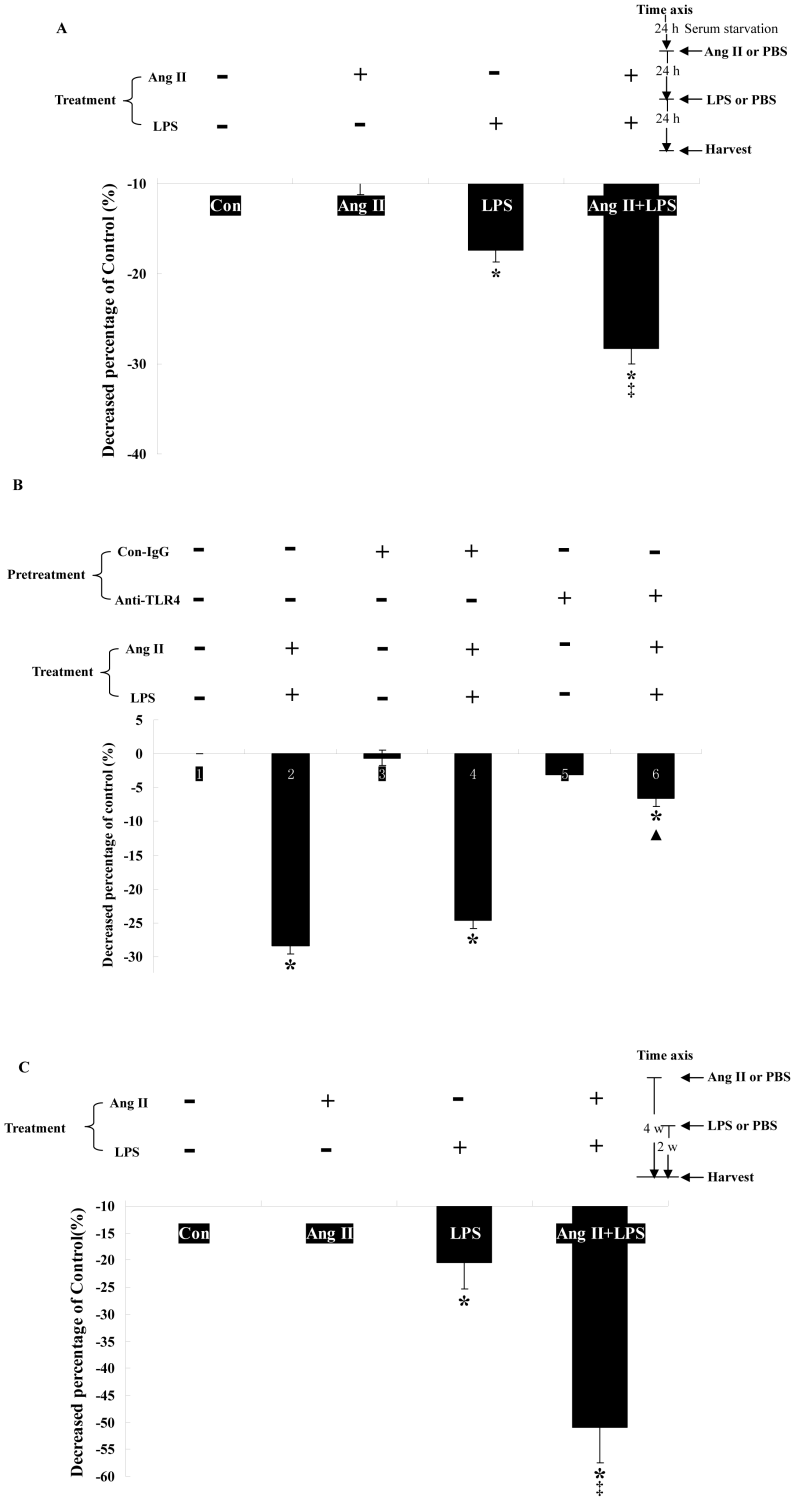
Ang II enhances down-regulation of *BAMBI mRNA* induced by LPS-TLR4 signal transduction. T6 cells and rat livers were harvested after treatment under conditions described before. Total RNA was analyzed for levels of *BAMBI mRNA* using real-time qRT-PCR, and data were normalized to *GAPDH mRNA* levels. Each data point represents means ±SE (n = 3). Data are representative of 3 experiments. A: T6 cells *in vitro*. B: Before Ang II and LPS combined treatment, anti-TLR4 neutralization antibody (1 mg/ml), control IgG2a (1 mg/ml) or PBS were used. C: primary HSCs isolated from livers of treated rats. * *p*<0.05, the levels of *BAMBI mRNA* in the cells of the LPS group or the Ang II+LPS group are lower than that of control group. ‡ *p*<0.05, shows a synergistic effect between Ang II and LPS on the effect of down-regulating *BAMBI mRNA* levels by factorial analysis. ▴ *p*<0.05, the *BAMBI mRNA* levels were higher than that of Ang II+LPS group.

Ekihiro S, et al reported that LPS induces HSC down-regulation of *BAMBI mRNA* expression through TLR4 [22]. Therefore, the variation in *BAMBI mRNA* expression may be used as an index of HSC LPS-TLR4 signaling. We used the degree of *BAMBI mRNA* down-regulation to estimate if the activity of the LPS-TLR4 signaling pathway was increased after TLR4 up-regulation induced by Ang II. The *Bambi mRNA* expression is shown not to be down-regulated by Ang II (10^−6^ mol/L), although it was by LPS (100 ng/ml) (down-regulated 17.3 ± 1.37%). Probably because of the low-level of HSC surface TLR4 expression without Ang II stimulation (Fig. 4 A-D). The expression level of *Bambi mRNA* was decreased 28.3 ± 1.74% by consecutive stimulation to Ang II (10^−6^ mol/L) and LPS (100 ng/ml). These values are higher than for a single stimulation by LPS, and a synergistic effect between them is shown by factorial analysis (F = 35.875, P<0.05) (Fig. 5A). The same conclusion is reached by using primary HSCs isolated from livers of treated rats (Fig. 5C). We also used anti-TLR4 neutralization antibody to block the interaction between LPS and TLR4. Then, the down-regulation of *Bambi mRNA* induced by Ang II and LPS combined stimulation was inhibited (Fig. 5B). These findings indicate that Ang II doesn't only induce Tlr4 expression, but also enhances the activity of HSC LPS-TLR4 signal pathway.

## Discussion

**Figure 6:**
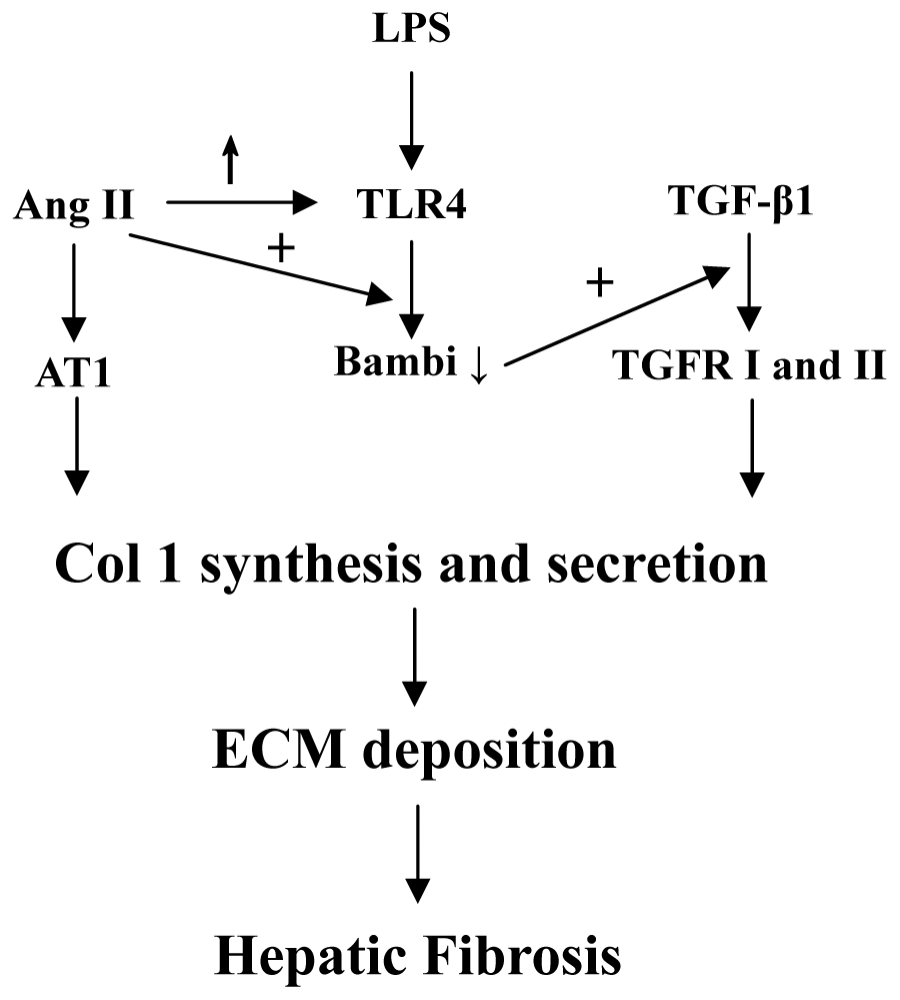
Crosstalk between Ang II and TGF-β1 is supported by the LPS-TLR4-BAMBI signal pathway in HSCs. Ang II induces Col 1 synthesis in, and secretion from, HSCs through AT1, so does TGF-β1 through TGFR I and II. LPS doesn't induce Col 1 synthesis and secretion in HSCs directly through TLR4, but LPS-TLR4 interaction down-regulates BAMBI expression, which is a TGF-β1 pseudo-receptor. Then the pro-fibrogenic function of TGF-β1 is enhanced. Ang II up-regulates TLR4 expression and enhances the activity of LPS-TLR4 signal pathway in HSCs, resulting in further down-regulation of BAMBI expression and up-regulated pro-fibrogenic function of TGF-β1. ↑ represents upregulation; ↓ represents downregulation; + represents enhancement.

TGF-β is considered as the most powerful mediator of HSC activation and plays a central role during hepatic fibrogenesis [22], [35], although *TGF-β* gene transgenic animals exhibit only limited fibrosis level [4]–[7]. The reason is that TGF-β needs to cooperate with other factors to induce fibrosis maximally. Here, experiments *in vitro* and *in vivo* confirmed that the maximal pro-fibrotic effect of TGF-β needs cooperation with Ang II and LPS (Fig. 6).

The LPS-TLR4 signal pathway activates HSCs and down-regulates BAMBI, with resulting enhanced sensitivity to TGF-β. It is indicated crosstalk between LPS and TGF-β on Col 1α production. However, single LPS pre-treatment followed by TGF-β1 treatment was not enough to obtain the synergistic effect (Fig. 1 and 2A p-t), because HSC only expresses minute amounts of TLR4 without Ang II (Fig.\ 3 and 4), while LPS then can't down-regulate BAMBI enough to sensitize HSCs in fully activated HSCs (T6 cells) and primary HSCs isolated from treated rat liver (Fig. 5). This phenomenon is not consistent with the previous work, in which only LPS treatment can down-regulate 80% of BAMBI expression in primary quiescent HSCs [22]. The reasons may be: First, Ekihiro S, et al treated primary quiescent HSCs with LPS for just 6 h, but we used 24 h in in-vitro experiment. In our opinion, HSCs were stimulated by LPS persistently during hepatic fibrogenesis. Thereby, our experiment comes more close to reality, in which the *BAMBI* mRNA level was only down-regulated around 20% by LPS alone. Second, Ekihiro S, et al isolated primary quiescent HSCs followed by magnetic antibody cell sorting with antibody against F4/80 antigen and CD11b-conjugated microbeads to remove Kupffer cells and macrophages. This procedure may activate primary quiescent HSCs and up-regulate TLR4 expression, because primary quiescent HSCs are easily to be activated even by culturing for certain time in dish [36]. Therefore, an efficient crosstalk must need up-regulation of TLR4. In this study, the up-regulation of TLR4 protein by Ang II was demonstrated both on T6 cells (Fig. 3) and primary HSCs in rat livers (Fig. 4). Furthermore, the function of Ang II on up-regulation of TLR4 was confirmed by ARB *in vitro* and *in vivo* (Fig. 3 and 4).

The *in vitro* and *in vivo* experiments showed that Ang II pre-treatment followed by exposure to TGF-β1 is not enough for a clear synergistic effect, although Ang II does up-regulate TLR4 successfully. The reason is that the TLR4 signal pathway isn't activated in the lack of its LPS ligand. Moreover, the BAMBI level can't be altered without LPS. Therefore, LPS is obligatory for the development of the full synergistic effect.

In conclusion, maximal pro-fibrotic effect of TGF-β requires up-regulation of TLR4 by Ang II and increased hepatic LPS levels. Only when Ang II, LPS and TGF-β together activate HSCs does a synergistic effect occur. In this process, Ang II is the initiator, the LPS-TLR4 signal pathway is the bridge and TGF-β is the executor, while none is dispensable. A variety of factors inducing chronic liver damage can activate HSCs and induce TGF-β and Ang II secretion. Ang II initially acts in the liver to up-regulate TLR4 expression in HSCs and constrict small blood vessels, which may result in portal hypertension. Then, perennial portal hypertension leads to increased intestinal permeability and bacterial translocation, resulting in accumulating LPS. Increased levels of LPS further activates HSCs through up-regulation of TLR4, initially induced by Ang II, and down-regulates BAMBI expression, resulting in sensitization of HSCs to TGF-β. Moreover, increased LPS also causes enhanced secretion of TGF-β and Ang II. Finally, a vicious circle forms (Fig. S1), being the reason that hepatic fibrosis is a refractory and progressive process causing cirrhosis or hepatic carcinoma, even though the pathogenic factors are removed. Therefore, removing pathogenic factor and anti-TGF-β strategies are not enough, but inhibition of RAS, TLR4 signaling and stabilization of intestinal permeability need to be added. Moreover, an anti-TGF-β therapy can't be used on long terms and in large quantity [8], [10]. Therefore, the combined therapy described above appears more promising.

Recently, Shirai et al. [36] reported that Ang II could up-regulate *TLR4* mRNA expression in activated rat HSCs by culturing on plastic plates for 7 days. This is consistent with our observations both on rat HSC cell line and primary HSCs, in which TLR4 protein levels were assessed by western blotting *in vitro* and immunofluorescence *in vivo*. Shirai et al. also suggested that the interactive effect between Ang II and LPS-TLR4 played a pivotal role in liver fibrosis development in nonalcoholic steatohepatitis, which may be through activation of myeloid differentiation factor 88 (MyD88), NF-κB and TGF-β expressions. While, our data showed that Ang II, LPS and TGF-β1 showed synergistic effects on Col 1α production in HSCs, mainly because that Ang II up-regulated HSC TLR4 expression and enhanced down-regulation of BAMBI caused by LPS, resulting in enhanced sensitivity to TGF-β.

Unfortunately, we can only demonstrate synergistic effects between Ang II, LPS and TGF-β followed the forward direction, in which stimulation by Ang II, LPS and TGF-β are induced sequentially, but not from the reverse, in which ARB, TLR4 knockout/TLR4 neutralization, or inhibition of BAMBI down-regulation, are used. In the latter case, the synergistic effect is certain to be inhibited because of blockage of their own fibrogenic effect, but not interfering with the crosstalk. Any technique that just inhibits the synergistic effects, but not prevents the basic pro-fibrotic effect of Ang II, LPS or TGF-β is not yet available. TLR4 knockout/TLR4 neutralization can be used as an example. Besides by down-regulation of BAMBI, the pro-fibrotic mechanisms of TLR4 signaling in HSCs are complex [37]: 1. LPS-TLR4 signal pathway induces chemokine secretion, chemokine receptor and adhesion molecule expression [22], [38]-[41]. 2. LPS regulates Col 1α-related microRNA through TLR4 [42]. 3. TLR4 signaling promotes the migration of liver sinusoidal endothelial cells and angiogenesis through HSC-derived fibronectin [43]. Therefore, liver fibrogenic susceptibility of rats, in which TLR4 signaling is interrupted, must be lower than that of wild type animals, but this may not be the case because of the crosstalk bwtween Ang II and TGF-β1 might have been cut off.

All kinds of pro-fibrotic factors exist in the fibrotic liver, and HSCs express receptors of pro-fibrotic factors simultaneously. Crosstalks between them must exist during the progress of liver fibrosis such as Ang II, LPS and TGF-β1. In fact, researchers have paid attention to the synergistic effects between pro-fibrotic factors for long time. Massimo P., et al. found that TGF-β enhanced DNA synthesis and HSCs proliferation induced by PDGF [44], and Breitkopf K., et al. reported that TGF-β1 induced *PDGF-AA* and *-BB mRNA* expression in HSCs [13]. Direct evidence showed that Ang II up-regulated the level of *TGF-β1* mRNA in HSCs [45]. All these facts prove that the interactive effects between pro-fibrotic factors are universal phenomenon like a network. Here we summarize the identified crosstalks between different pro-fibrotic factors known so far in Fig. S2. The table on the top right corner lists candidate factors, which may perform crosstalk in HSCs during hepatic fibrosis. We believe this to be only a small step of forward in our ongoing studies on liver fibrosis, but one giant leap in understanding the network mechanism of hepatic fibrosis.

## Supporting Information

Figure S1 Autogenous long-standing vicious circle formed by Ang II, LPS and TGF-β1 in HSCs during the progress of liver fibrosis.
(DOC)

Figure S2 The interactive effect between hepatic fibrosis-related factors in HSCs. 1. Ang II promotes the pro-fibrogenic effect of TGF-β1 through the AT1-TLR4-Bambi axis. 2. TGF-β enhances DNA synthesis and HSCs proliferation induced by PDGF. 3. TGF-β1 induces PDGF-AA and -BB mRNA expression in HSCs. 4. Ang II up-regulates TGF-β1 mRNA level in HSCs. 5. The list contains partly identified fibrotic and anti-fibrotic factors expressed in HSCs that may become another crosstalk pair. Our work is summarized in bold.
(DOC)
