# Supplementary material for: Angiotensin II Facilitates Fibrogenic Effect of TGF-β1 through Enhancing the Down-Regulation of BAMBI Caused by LPS: A New Pro-Fibrotic Mechanism of Angiotensin II
Source: PLoS One. 2013 Oct 14;8(10):e76289. doi: 10.1371/journal.pone.0076289 (PMC3796560; doi:10.1371/journal.pone.0076289)

Supplementary Fig. 1

Autogenous long-standing vicious circle formed by Ang II, LPS and TGF-β1 in HSCs during the progress of liver fibrosis.


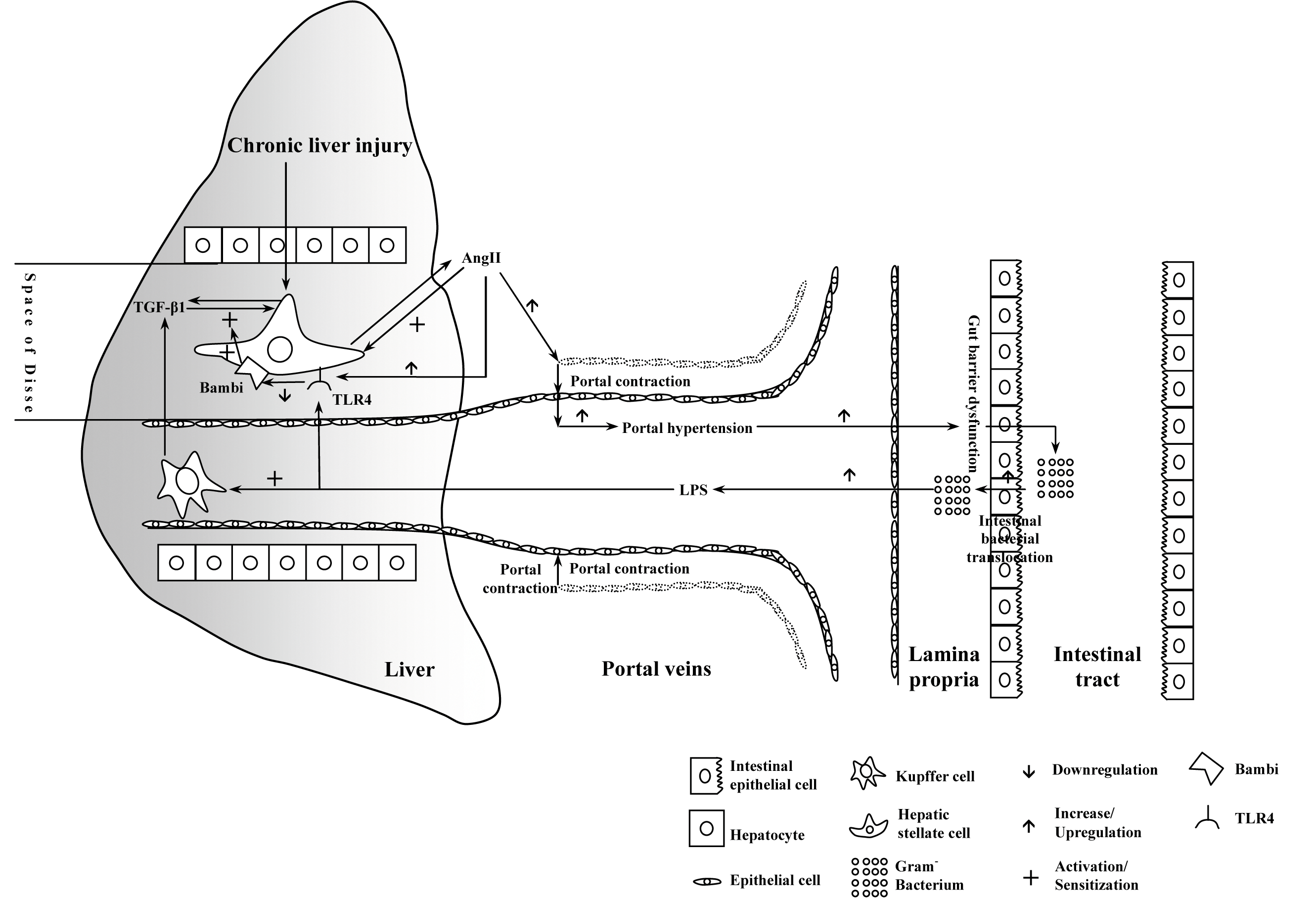

Supplement: Figure S1 — Autogenous long-standing vicious circle formed by Ang II, LPS and TGF-β1 in HSCs during the progress of liver fibrosis. (DOC) [file pone.0076289.s001.doc]
