# Supplementary material for: Angiotensin II Facilitates Fibrogenic Effect of TGF-β1 through Enhancing the Down-Regulation of BAMBI Caused by LPS: A New Pro-Fibrotic Mechanism of Angiotensin II
Source: PLoS One. 2013 Oct 14;8(10):e76289. doi: 10.1371/journal.pone.0076289 (PMC3796560; doi:10.1371/journal.pone.0076289)

Supplementary Fig. 2

The interactive effect between hepatic fibrosis-related factors in HSCs. 1. Ang II promotes the pro-fibrogenic effect of TGF-β1 through the AT1-TLR4-Bambi axis. 2. TGF-β enhances DNA synthesis and HSCs proliferation induced by PDGF. 3. TGF-β1 induces PDGF-AA and -BB mRNA expression in HSCs. 4. Ang II up-regulates TGF-β1 mRNA level in HSCs. 5. The list contains partly identified fibrotic and anti-fibrotic factors expressed in HSCs that may become another crosstalk pair. Our work is summarized in bold.


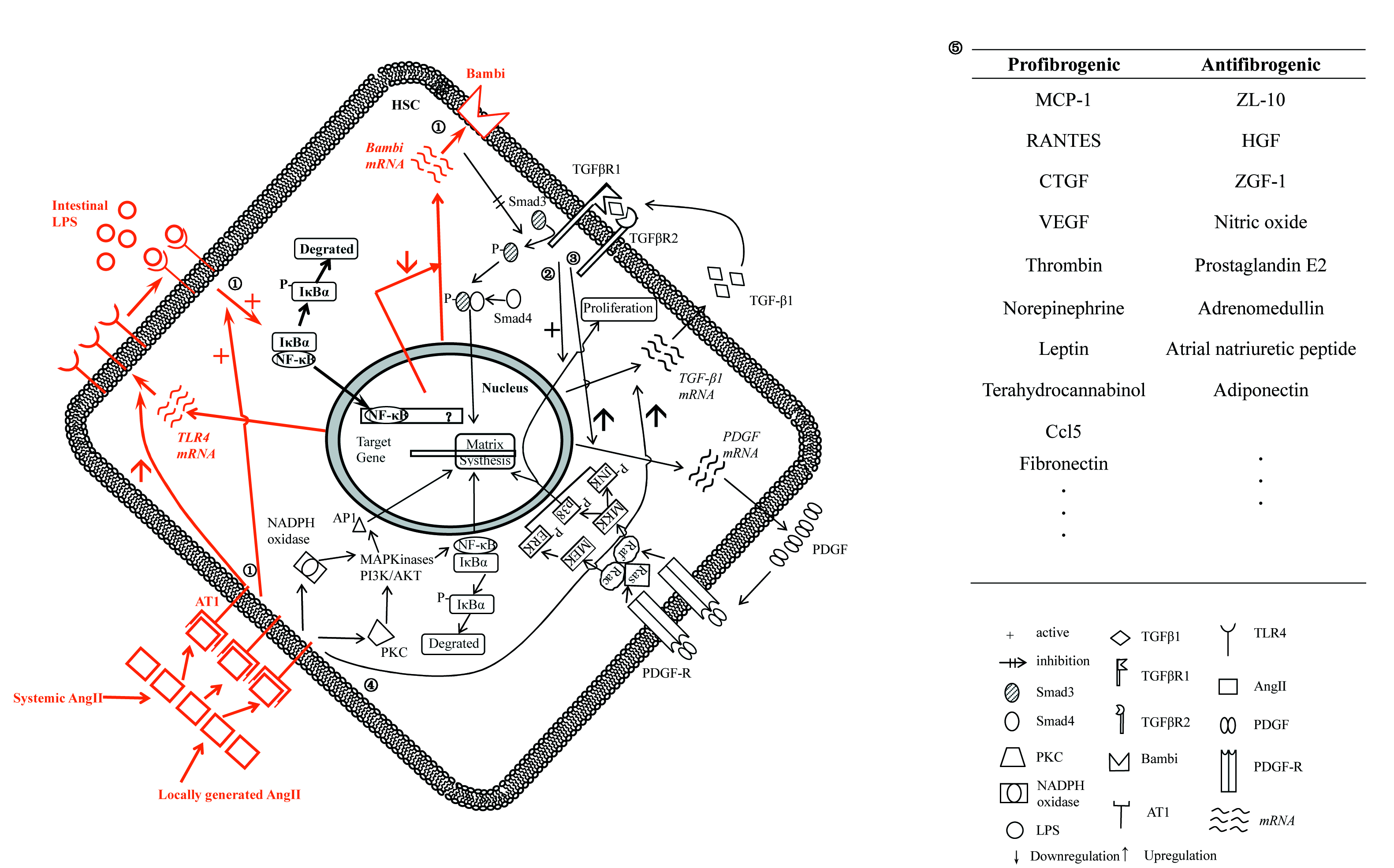

Supplement: Figure S2 — The interactive effect between hepatic fibrosis-related factors in HSCs. 1. Ang II promotes the pro-fibrogenic effect of TGF-β1 through the AT1-TLR4-Bambi axis. 2. TGF-β enhances DNA synthesis and HSCs proliferation induced by PDGF. 3. TGF-β1 induces PDGF-AA and -BB mRNA expression in HSCs. 4. Ang II up-regulates TGF-β1 mRNA level in HSCs. 5. The list contains partly identified fibrotic and anti-fibrotic factors expressed in HSCs that may become another crosstalk pair. Our work is summarized in bold. (DOC) [file pone.0076289.s002.doc]
